# Quantification of periaortic adipose tissue in contrast-enhanced CT angiography: technical feasibility and methodological considerations

**DOI:** 10.1007/s10554-022-02561-8

**Published:** 2022-02-26

**Authors:** Apostolos T. Mamopoulos, Patrick Freyhardt, Aristotelis Touloumtzidis, Alexander Zapenko, Marcus Katoh, Gabor Gäbel

**Affiliations:** 1 Faculty of Medicine, Saarland University, Kirrbergerstrasse 100, D-66421 Homburg/Saar, Germany; 2 Present Address: Department of Vascular Surgery, HELIOS Klinikum Krefeld, Lutherplatz 40, 47805 Krefeld, Germany; 3 Institute for diagnostic and interventional Radiology, HELIOS Klinikum Krefeld, Lutherplatz 40, 47805 Krefeld, Germany; 4 School of Medicine, Faculty of Health, University Witten/Herdecke, Alfred-Herrhausen-Straße 50, 58455 Witten, Germany

**Keywords:** Periaortic fat tissue, Abdominal aortic aneurysm, Periaortic adipocytes, Aneurysm progression

## Abstract

To examine the feasibility of the quantification of abdominal periaortic fat tissue (PaFT) (tissue within − 45 to − 195 HU) in enhanced CT-angiographies compared to unenhanced CT-scans and identify methodological issues affecting its clinical implementation. Using OsirixMD, PaFT volume and mean HU value were retrospectively measured within a 5 mm periaortic ring in paired unenhanced and enhanced abdominal aortic CT-scans. The correlation between PaFT values was examined in a derivation cohort (n = 101) and linear regression analysis produced correction factors to convert values from enhanced into values from unenhanced CTs. The conversion factors were then applied to enhanced CTs in a different validation cohort (n = 47) and agreement of corrected enhanced values with values from unenhanced scans was evaluated. Correlation between PaFT Volume und Mean HU from enhanced and unenhanced scans was very high (r > 0.99 and r = 0.95, respectively, p < 0.0001 for both). The correction factors for PaFT Volume and Mean HU were 1.1057 and 1.0011. Potential confounding factors (CT-kilovoltage, slice thickness, mean intraluminal contrast density, aortic wall calcification, longitudinal variation of intraluminal contrast density, aortic diameter) showed no significant effect in a multivariate regression analysis (p > 0.05). Bland-Altman analysis of corrected enhanced and unenhanced values showed excellent agreement and Passing–Bablok regression confirmed minimal/no residual bias. PaFT can be quantified in enhanced CT-angiographies very reliably. PaFT Volume scores are very consistently slightly underestimated in enhanced scans by about 10%, while the PaFT Mean HU value remains practically constant and offers distinct methodological advantages. However, a number of methodological issues remain to be addressed.

**Supplementary Information:**

The online version contains supplementary material available at 10.1007/s10554-022-02561-8.

## Introduction

Periadventitial adipose tissue functions as a fourth arterial layer secreting vasoactive, often pro-inflammatory, substances [1], with inflammation playing a critical role in the pathophysiology of abdominal aortic aneurysms (AAAs) [2]. Adventitial fibroblasts produce cytokines and enzymes causing extracellular matrix degradation and neovascularization leading to medial degeneration and adventitial collagen degradation implicated in AAA progression [3], while an increased number of inflammatory cells has been found in tissue surrounding AAAs [4–6]. Periaortic fat tissue (PaFT) is histologically a distinct entity from retroperitoneal tissue, comprising small, white, dense adipocytes with a distinct, extremely rich vascular bed [7]. Furthermore, PaFT shows adventitial encroachment into the adjacent vessel, and is interspersed with vasa vasorum [8, 9], making PaFT a good candidate for paracrine signaling [10] and bidirectional communication with the aortic wall [11, 12]. Moreover, pronounced adventitial adipocyte-aggregates in AAAs [13], increased adipogenic potential of AAA adventitial mesenchymal cells and observed enrichment of adipocyte-related genes in ruptured AAA, support an association between increased fatty adventitial degeneration and rupture risk [13].

Recently, a direct link between the histological and the CT-imaging characteristics of perivascular adipose tissue was demonstrated in vivo and in vitro [14]. Increased PaFT Volume likely represents a higher concentration of periaortic adipocytes and, thus, a greater inflammatory effect, but it may also be partially caused by locally increased number of fat pixels in the aortic wall itself. Furthermore, since adipocyte lipid content is the main component of PaFT, larger and more numerous adipocytes have a higher proportion of lipid phase (adipocytes) compared to aqueous phase (extracellular space), leading to more negative attenuation values [14].

Therefore, PaFT measurement could be of clinical interest for AAA prognosis, which is currently based on the maximum diameter. OsiriXMD® is a commercially available software with available tools for the measurement of PaFT. PaFT quantification has so far been mostly performed in unenhanced CT- scans correlating PaFT to coronary and peripheral vascular disease or metabolic risk. On the other hand, exploring a potential association of PaFT with the pathophysiology of AAAs requires PaFT quantification in CT angiographies, since in clinical practice AAAs are preoperatively usually imaged with enhanced CTs omitting the non-enhanced phase [15]. While the issue has been raised when quantifying pericoronary fat tissue [14], no systematic assessment has been made to address the impact of enhancement on PaFT values, and the potential interference of intraluminal contrast medium in PaFT identification and quantification, as it happens e.g. in the quantification of aortic calcification [16]. As contrast medium is strongly attenuating in CT-imaging, it can thereby cause severe metal-like artifacts that may impair assessment of surrounding perivascular structures, including fat tissue [17, 18]. Another potential cause of interference could be an early enrichment of the very richly vascularized PaFT.

Therefore, the aim of this study was to evaluate the effect of contrast medium and the agreement between PaFT measurements with and without contrast medium and to identify and possibly address any related methodological issues. The results of the study are essential to establish the methodology of PaFT quantification, enabling a further examination of the potential prognostic value of PaFT for aortic disease and its future clinical applications.

## Methods

### Study design

The retrospective nature of the study did not necessitate an a priori calculation of a sample size, since the statistical significance of the results can be determined by the confidence levels of the resulting outcomes. Sample size was determined based on existing literature on PaFT quantification [19] and contrast medium interference [20]. Consecutive abdominal or thoracoabdominal CT-scans performed in our Radiology Department between 05.12.2018 and 04.07.2019 (for the derivation study) and 01.01.2020–30.03.2020 (for the validation study) were reviewed for inclusion. Included were CT-scans containing the entire infrarenal aorta with at least one native (unenhanced) and one arterial (enhanced) phase, which were paired with identical slice thickness/increment (to exclude their effect on volume reconstructions) and CT-tube voltage (to exclude its effect on tissue attenuation). Current-exposure time product (mAs) was not considered since it does not affect tissue attenuation values.

### Exclusion criteria

Surgically or endovascular treated aortas, peri-/aortitis or inflammatory AAA, ruptured AAAs, intra- or paraaortic foreign bodies (stents, coils, embolizing factors, cava filters), inadequate aortic imaging (artefacts by spinal osteosynthesis) and for the arterial phase, an intraluminal standard deviation > 35 HU indicative of very high image noise. Patients with large AAAs or low cardiac output (varied contrast distribution) were not excluded.

### Imaging protocols

Images were obtained using a 256-detector (Philips, Brilliance iCT) and a 64-detector (Philips, Ingenuity) multiscanner with 2 × 128 × 0.625 mm and 64 × 0.625 mm detector collimation and 0.27 and 0.42 s gantry rotation, respectively. The image slice/increment were identical in both phases, usually 3 mm/2 mm. Examination-pairs with a slice thickness/increment of 5 mm/4 mm were also included. (Table 1) The kilovoltage setting was typically 120 kV for both phases. Paired examinations with 100 kV and 130 kV were also included. Tube current modulation was applied to all examinations. For all of the enhanced CTs, 100 ml contrast medium (Accupaque 350) was delivered at 4 ml/sec by an automated injection driver system, triggered when a threshold of 150 HU was reached at the center of the aorta. A sharp (C) reconstruction kernel was used for both phases.

**Table 1:** Imaging parameters and PaFT volume and mean HU values, derivation study (n = 101)

Mean age (n = 101)/years	71.8 (± 10.6) [48–94]
Sex—male (%)	62 (61.4)
Mean intraluminal attenuation, arterial phase/HU	315.9 (± 82.3) [195–612]
Size of intraluminal contrast sample-ROI/mm	8 mm, n = 41
	10 mm, n = 39
	12 mm, n = 21
Slice thickness/mm	3 mm, n = 96
	5 mm, n = 5
CT tube kilovoltage/kV	100 kV, n = 14
	120 kV, n = 86
	130 kV, n = 1
Mean aortic diameter/mm	
Total, n = 101	26.9 (± 14.6) [15.6–110.5]
Non-AAAs (< 30 mm), n = 83	22.3 (± 2.7) [15.6–29.4]
AAAs (> 30 mm), n = 14	54.2 (± 24.3) [31–110.5]
Median aortic volume/*mean*/cm^3^	27.2 (14) [10.9–749]/*48.9*
Median periaortic volume/*mean*/cm^3^	62.1 (22.4) [29.9− 906.1]/*86.7*
Median periaortic Ring volume/*mean*/cm^3^	33.8 (9.2) [18.2 − 156.8]/*37.8*
Median difference arterial PaFTVol-native PaFTVol/cm^3^	− 1.75 (1.1) [− 10.3 to 0.4]/*− 1.96*
Median difference arterial PaFTVol-native PaFTVol/%	− 10.9 (9.1) [− 40.7 to 59.3]/*− 12.56*
Median longitudinal intraluminal attenuation variability/%	4.46 (4.1) [5.2–82.6]/*6.1*
Median intraluminal SD, arterial phase/*mean*/HU	21.7 (5.1) [13.8–38.7]/*22.2*

	Mean PaFT volume/cm^3^	Mean [PaFTVolume]*	Mean of PaFT-Mean HU
Native	17.6 (± 13.68) [0.34–105.25]	0.467 (± 0.221) [0.006–0.882]	− 77.0 (± 9.7) [− 101.9 to − 42.9]
Arterial	15.65 (± 12.66) [0.33–94.9]	0.414 (± 0.211) [0.006–0.829]	− 76.8 (± 10.2) [− 104.1 to− 29.8]

*[]: PaFT Volume adjusted for periaortic Ring Volume: [PaFTVolume] = PaFTVolume/Periaortic Ring Volume

### Processing in OsiriXMD and PaFT measurement

**Fig. 1:**
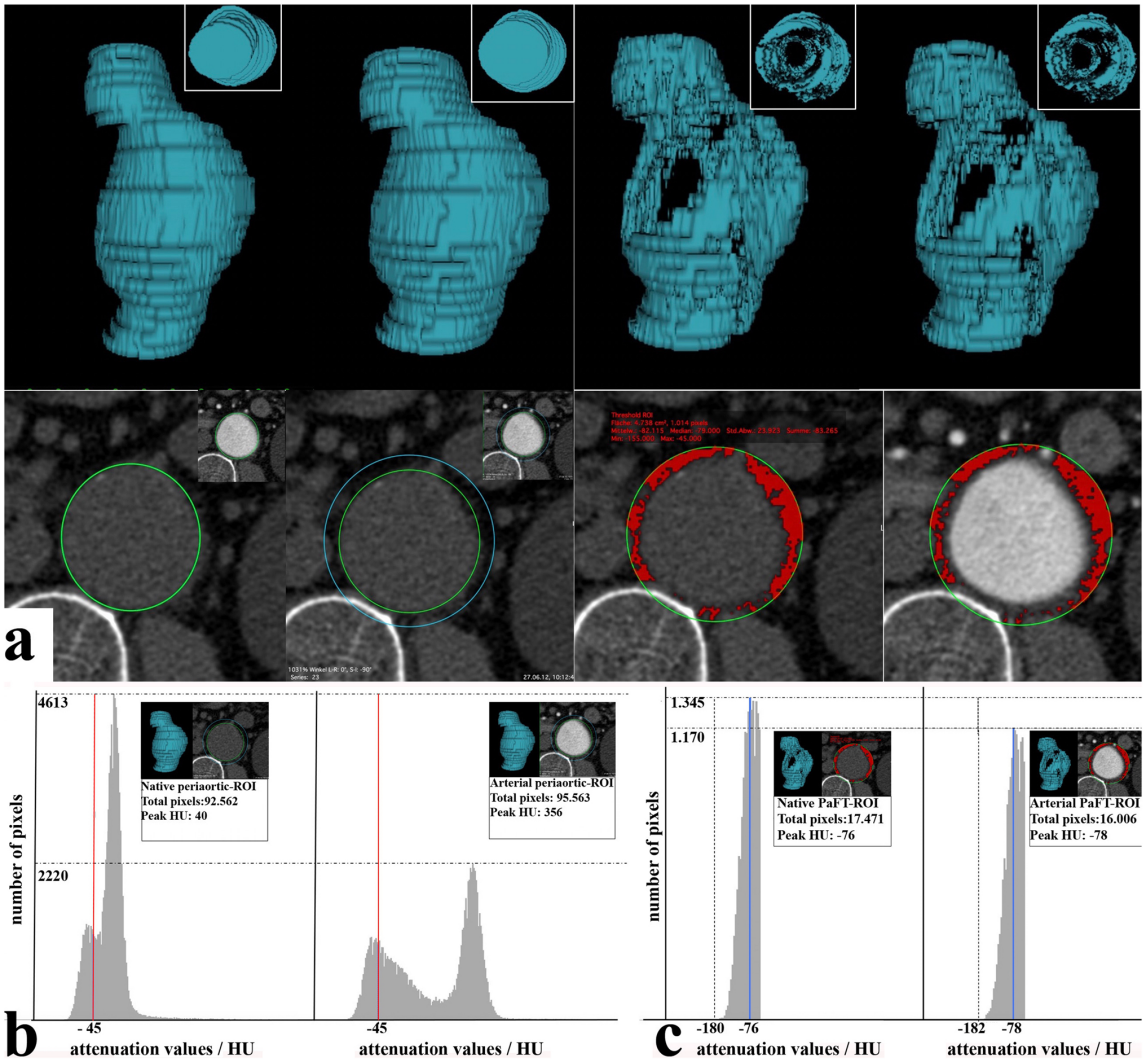
**a** Summary of PaFT measurements. The top images show the reconstructed volumes measured (from left to right: aortic Volume, periaortic Volume, native PaFT Volume and arterial PaFT Volume. Note that the top views of the 3D reconstructed PaFT volumes show the inside of the cylinder containing different aortic contours and not fat-containing voxels within the aortic disc). The bottom images show the respective ROIs used for the reconstructed volumes (from left to right: native aortic volume, native and aortic periaortic volumes, native PaFT volume and arterial PaFT volume). **b** Distribution of pixel HU values within the periaortic ROI in the native and arterial scans (the red line represents the upper threshold for the fat tissue containing pixels with values − 45 HU to − 195 HU). In the native phase pixel HU values peak at about 40 HU. In the arterial phase pixel values within the identical periaortic ROI peak at about 356 HU. The distribution of pixels with values below − 45 HU shows few changes. **c** Distribution of pixel HU values within the PaFT-ROI in the native and arterial scans (the blue line indicates the range of HU values containing the peak HU value). The distribution of HU value frequencies changes slightly between the native and arterial phase. The total number of fat tissue-containing pixels are slightly reduced (from 17 471 in the native to 16 006 in the arterial phase) and so does the total Volume of ROIs with pixels with values − 45 to − 195 HU. The peak HU value of the PaFT-ROIs remains relatively constant. The objective of our study is to further examine these relationships in a statistically significant sample

Firstly, we selected an aortic and periaortic region-of-interest (ROI) in every axial image between the most distal renal artery and the aortic bifurcation. Based on published protocols [19, 21–23] we measured PaFT within a 5 mm-wide periaortic circular ring area, introduced a ROI co-centered with the infrarenal aortic disc in all unenhanced images, and modified it to match the outer circumference of the aortic wall. The periaortic ROIs were then established, by extending the aortic ROIs by 10 mm in both axes and were copied and transferred to identical axial images of the enhanced series. (Fig. 1a) There are a number of ways to deal with non- circular aortic discs in axial images. (Online Resource 1). For non-circular aortic discs, we firstly traced an oval aortic ROI following the aortic contour as closely as possible and then extended this aortic ROI by 10 mm in both diameters. We included non-circular aortic discs in our model, since they were identical in both CT phases and thus inconsequential when comparing areas between CT-phases. The Aortic Volume (AVol) resulted from volume reconstruction of all the aortic ROIs in the unenhanced series. The Periaortic Volume (PaVol) resulted from volume reconstruction of all periaortic ROIs in the enhanced and unenhanced series, which was identical in both phases. The Periaortic Ring Volume (PaRVol), equal to the volume of periaortic tissue within 5 mm of the aortic wall, was defined by subtracting the AVol from the PaVol. (Fig. 1a) A review of existing literature (Online Resource 2) showed that almost all studies (including the three most relevant studies in the infrarenal aorta [19, 21, 22] used a density range of − 195 to − 45 HU to detect fat-containing voxels. After that, we set all pixels outside the periaortic ROI to zero because the software cannot be restricted by pre-existing ROIs and would include in the measurement all voxels with HU values between − 45 and − 195 HU irrespective of location. We then selected all voxels with attenuation values of − 195 to − 45 HU within the periaortic ROIs in the unenhanced and enhanced phase. The GlobalThresholding Plugin [Rene Laqua (2016) GlobalThresholding,v1.0, 10.5281/zenodo.208170] greatly simplifies processing by creating a separate ROI in every axial image, which includes all voxels with signal densities in the selected range. Before applying it, we performed a preliminary validation of it, confirming the validity of its created ROIs compared to the organic function of the OsirixMD platform. (Online Resource 3).

These ROIs were then volume-reconstructed to give the total volume of voxels within the − 195 to − 45 HU range in both the unenhanced and arterial phases (PaFTVol). (Fig. 1a) as well as the PaFT Mean HU attenuation value (PaFTmeanHU) and its standard deviation (SD). (Fig. 1b, c) This approach assumes that all relevant voxels (− 195 to − 45 HU) in the periaortic ROI are located strictly within the 5 mm-wide periaortic ring and not within the unenhanced aortic disc, which was proven to be the case in our study. (Online Resource 1) The maximum aortic diameter was defined as the shorter axis of the largest aortic disc. Since the amount of PaFT depends on the amount of total periaortic volume, the PaRVol was used then to adjust the PaFT volume for the size of the aorta and consequently size of PaVol, in order to calculate a PaFT-“ratio” [PaFTVol]= PaFTVol/PaRVol. The impact of intraluminal contrast medium was examined by setting an intraluminal sample ROI and measuring the mean contrast HU value and its standard deviation. To examine the impact of lateral contrast dispersion, three different sample ROI-sizes were applied (8, 10 and 12 mm). To account for longitudinal contrast dispersion, an average contrast mean HU value was calculated from three measurements (infrarenal, mid-aortic and bifurcation level) and a longitudinal contrast variability was defined as: (maximum contrast HU)–(minimum contrast HU)/(average contrast mean HU) and examined in multi-regression analysis. Additionally, a modified Agatston score was measured (using the Calcium Score Plugin in the unenhanced series with a calcium detection threshold at 130 HU). Our preliminary analysis showed that lateral contrast dispersion would not be an issue, whereas longitudinal variability in larger AAAs (> 100 cm^3^) could be significant (Online Resources 4, 5). As a result, we also performed a subgroup analysis excluding larger AAAs (> 55 mm). PaFT measurements were performed by a radiologist and an endovascular surgeon, each with more than 10 years of experience in aortic CT imaging analysis.

## Statistical analysis

Continuous variables were expressed as mean (standard deviation) [range] when normally distributed and median (interquartile range) [range] when skewed. Categorical variables were expressed as percentages. Based on similar studies comparing the effect of contrast medium on specific periaortic tissue [20] we followed the following methodology. In the derivation study the correlation between PaFT Volume/mean HU value in the unenhanced and arterial phase was examined and univariate linear regression was performed, in order to define a conversion factor. Potential confounding factors were further examined as independent co-variables in multivariate regression analysis. Variables were introduced in the model if they showed statistic relevance (p < 0.05). The conversion factor was then applied in the validation study to correct PaFT Volume/mean HU from enhanced CTs. In this context, comparing either total PaFT Volumes or PaFT-“ratio” yields the same result, since the latter is equal to the former divided by the PaRVol, which is identical in both CT phases. In the validation study, agreement between corrected enhanced and unenhanced PaFT Volumes/mean HU was examined with Bland-Altman plots and Passing-Bablok regression. Average values in the pooled non-AAA and AAA group from both phases were compared with the Mann-Whitney test. A two-tailed p < 0.05 was considered statistically significant. Analysis was performed on MedCalc® (Belgium). Intraobserver (after 1 year) and interobserver variability were examined in 20 data sets with the Intraclass correlation coefficient (ICC).

## Results

### Normality of distribution

For both [PaFTVolume] and mean PaFT HU value, normality of sample distribution was accepted by both Shapiro–Wilks and Kolmogorov–Smirnov tests and visually confirmed in histograms and QQ plots. (Online Resource 6a, b)

### Derivation study

From initially 114 selected data sets, during post-processing another 13 data sets were deemed incompatible with further processing because of slice mismatch between native and arterial phase (n = 1), incomplete imaging of the abdominal aorta (n = 1), uneven slice thickness precluding volume reconstruction (n = 11). Finally, 101 paired data sets were included in the statistical analysis. (Table 1)

#### Correlation

*PaFT Volumes* in enhanced CT-scans showed excellent correlation with scores from unenhanced scans with a Pearson’s coefficient r = 0.995 (p < 0.0001), 95% CI [0.993–0.997]. The correlation did not change when large AAAs (> 55 mm) were excluded from analysis: r = 0.993 (p < 0.0001), 95% CI [0.990–0.996]. *Mean PaFT HU values* in enhanced CT-scans also showed a very high correlation with values from unenhanced scans with a Pearson’s coefficient r = 0.952 (p < 0.0001) 95% CI [0.929–0.968]. The correlation did not change when large AAAs (> 55 mm) were excluded from the analysis r = 0.953 (p < 0.0001), 95% CI [0.930–0.969].

#### Linear regression

**Fig. 2:**
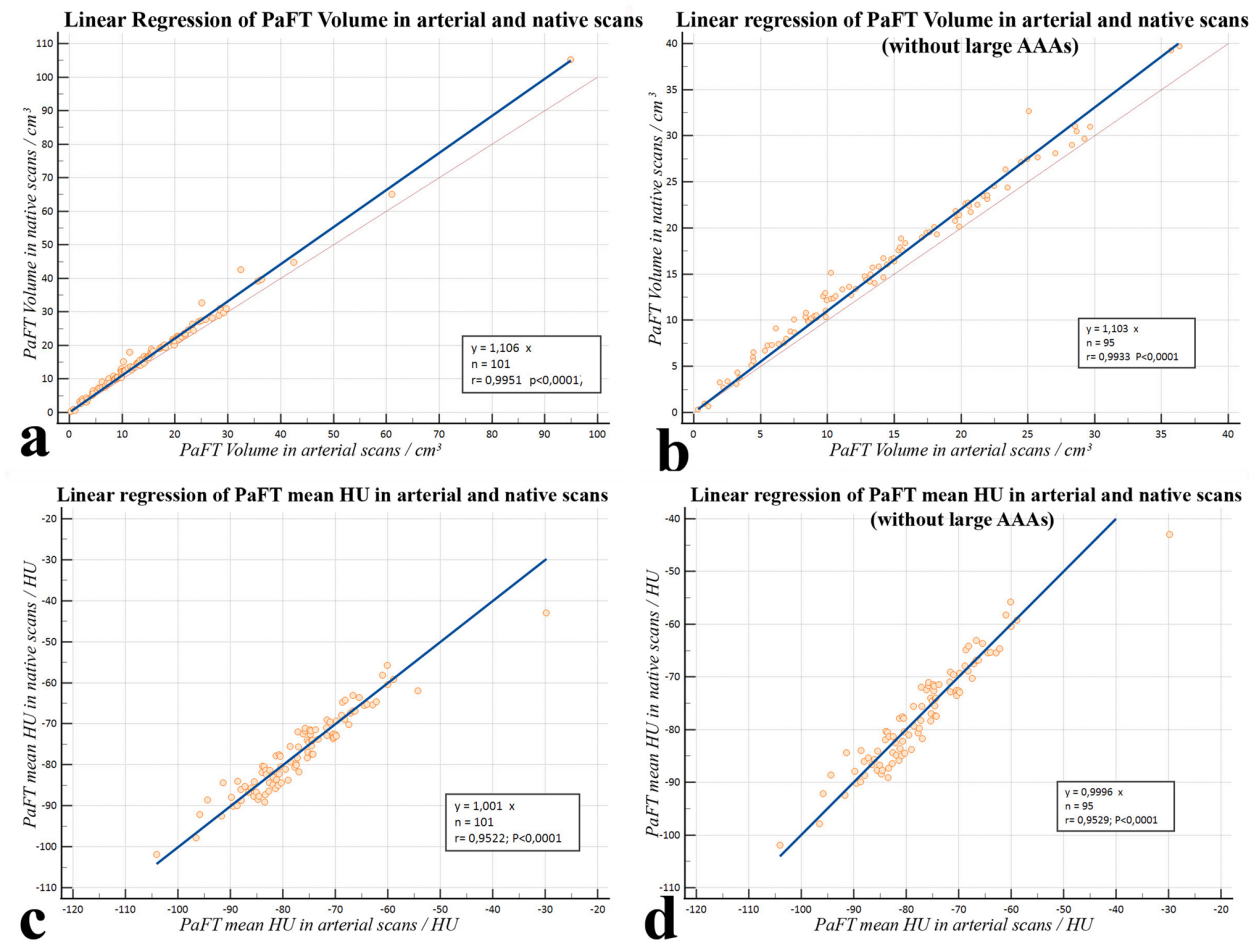
Univariate linear regression in the derivation study. For PaFT Volume including (**a**) and excluding (**b**) large AAAs and for the mean PaFT HU value including (**c**) and excluding (**d**) large AAAs the slope of the line of best fit was the correction factor for conversion of arterial to native PaFT values

Univariate linear regression through the origin produced a regression coefficient of 1.1057, (p < 0.0001), 95% CI [1.092–1.120], t = 154.950 for PaFT Volume and 1.0011, (p < 0.0001), 95% CI [0.993–1.009], t = 249.795 for mean PaFT HU value (Fig. 2).

Multivariate regression with native PaFT values as the dependent variable and possible confounding factors (along with arterial PaFT values) as independent variables showed that for both PaFT Volume and PaFT mean HU value none of the following factors had a significant effect (p < 0.05) on the correlation of enhanced with unenhanced PaFT scores: aortic wall calcification, mean intraluminal contrast intensity, maximum aortic diameter, slice thickness, CT-tube voltage, image noise (mean SD in arterial phase) and longitudinal variability of intraluminal contrast intensity. (r^2^ > 0.99 for the PaFT Volume and r^2^ = 0.94 for the Mean HU model, p < 0.0001 both). Subsequently, we formulated the following conversion equations to be applied in the validation study: *corrected PaFT Volume= 1.1057 x arterial PaFT Volume* and *corrected PaFT mean HU value= 1.0011 x arterial PaFT*. **(**Online Resources 7, 8)

### Validation study

From 53 initially selected data sets, during post-processing another six data sets were deemed incompatible with further processing because of slice mismatch between native and arterial phase (n = 1), incomplete imaging of the abdominal aorta (n = 2), uneven slice thickness precluding volume reconstruction (n = 3). Finally, 47 paired data sets were included in the statistical analysis (Table 2). During statistical processing three outlier sets were found, with normal levels of noise in the native but very high noise levels in the arterial phase resulting in abnormally low PaFT Volumes and high PaFT Mean HU values. These cases were excluded from further validation.

**Table 2:** Imaging parameters and PaFT volume and mean HU values, validation study (n = 47)

Mean age/years	71.4 (± 10.65) [48–93]
Sex—male (%)	29 (61.7)
Mean intraluminal attenuation, arterial phase/HU	312.7 (± 98.6) [127.5–605.5]
Size of intraluminal sample-ROI/mm	8 mm, n = 47
Slice thickness/mm	3 mm, n = 41
	5 mm, n = 6
CT tube kilovoltage/kV	100 kV, n = 1
	120 kV, n = 42
	130 kV, n = 4
Mean aortic diameter/mm	
Total, n = 47	25.8 (± 12.1) [18.4–90.7]
Non-AAAs (< 30 mm), n = 39	21.8 (± 2.5) [18.4–28.8]
AAAs (> 30 mm), n = 8	45.4 (± 20.2) [30.4—90.7]
Median aortic volume/*mean*/cm^3^	25.1 (15.1) [15.8–510.5]/*42.9*
Median periaortic volume/*mean*/cm^3^	56.3 (24.2) [37.8–646.97]/*78.3*
Median periaortic Ring volume/*mean*/cm^3^	31.3 (9.8) [21.98–136.5]/*35.5*
Median difference arterial PaFTVol-native PaFTVol/cm^3^	− 1.49 (1.65) [− 10.97 to 0.92]/*− 1.92*
Median difference arterial PaFTVol-native PaFTVol/%	− 13.4 (12.9) [− 65.9 to 5.8]/*− 16.2*
Median intraluminal SD, arterial phase/*mean*/HU	22.2 (5.6) [9.5–124.4]/*24.9*

	Mean PaFT volume/cm^3^	Mean [PaFTVolume]*	Mean of PaFT-Mean HU
Native	15.2 (± 11.7) [0.3–56.4]	0.413 (0.241) [0.02–0.906]	− 75.6 (± 9.0) [− 91.0 to − 59.1]
Arterial	13.3 (± 10.4) [0.26–45.4]	0.361 (± 0.223) [0.01–0.854]	− 74.0 (± 10.6) [− 91.0 to − 45.6]

*[] is the PaFT Volume adjusted for periaortic Ring Volume: [PaFTVolume] = PaFTVolume/Periaortic Ring Volume

The values in italics are the mean values given additionally to the median values. This is indicated in the left hand column as : Median aortic volume / mean (in italics)

**Fig. 3:**
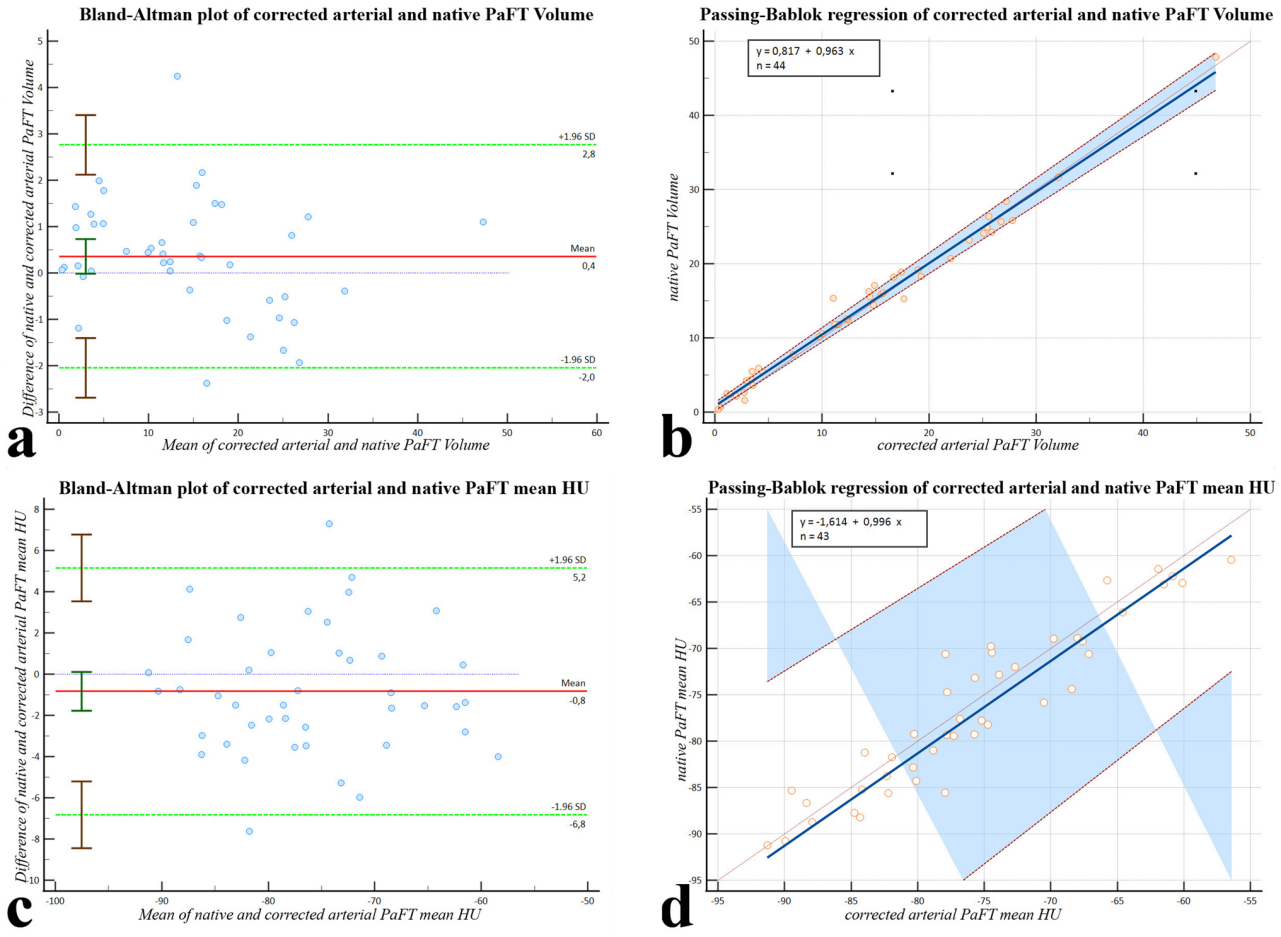
**a**, **b** Comparison of corrected arterial PaFT Volume and native PaFT Volume. The Bland–Altman plot on the left [mean difference 0.359 (− 0.0141 to 0.732), limits if agreement − 2.046 (− 2.688 to − 1.404) and 2.764 (2.121–3.406)] after removal of three outliers shows a good dispersion of values with only one value outside the LoA. The blue line of zero difference is inside the bar representing the 95% CI of the mean difference. The Passing–Bablok plot on the right showed only minimal systematic bias. The Cusum test (p = 0.59) and Spearman coefficient (r = 0.983, p < 0.0001) showed no significant deviation from linearity. **c**, **d** Comparison of corrected arterial PaFT mean HU value and native PaFT mean Hu value. The Bland–Altman plot on the left [mean difference − 0.835 (− 1.775–0.106), limits if agreement − 6.823 (− 8.443 to − 5.204) and 5.154 (3.534–6.773)] after removal of three outliers shows a good dispersion of values with only one value outside the LoA. The blue line of zero difference is inside the bar indicating the CI of the mean difference. The Passing–Bablok plot on the right showed no significant proportional or systemic bias. The Cusum test (p = 0.56) and Spearman coefficient (r = 0.943, p < 0.0001) showed no significant deviation from linearity

*PaFT Volume*. Bland-Altman analysis resulted in a mean difference between native and corrected arterial values of 0.359 with a 95% confidence level including 0.0 [− 0.0141–0.732] indicative of no significant residual bias. The hypothesis of agreement (H0: Mean = 0) was accepted (p = 0.0589). Passing-Bablok regression model showed no proportional bias [slope B = 0.963 with 95% CI including 1.0 (0.923–1.009)] and only minimal systematic bias [intercept A= 0.817 with 95% CI just outside 0.0 (0.217–1.295)]. Mean values were 14.399 for the native and 14.041 for the corrected arterial cohort (Fig. 3a, b).

*Mean PaFT HU value*. Bland–Altman analysis resulted in a mean difference between native and corrected arterial values of − 0.835 with a confidence level including 0.0 [− 1.775–0.106] indicative of no significant residual bias. The hypothesis of agreement (H0: Mean = 0) was accepted (p = 0.0804). Passing-Bablok regression model showed no proportional bias [slope B = 0.996 with CI including 1.0 (0.889–1.118)] and no systematic bias [intercept A = − 1.614 with 95% CI including 0.0 (− 9.420–7.666)]. Mean HU values were − 76.538 for the native − 75.703 for the corrected arterial cohort (Fig. 3c, d).

### Secondary study

**Fig. 4:**
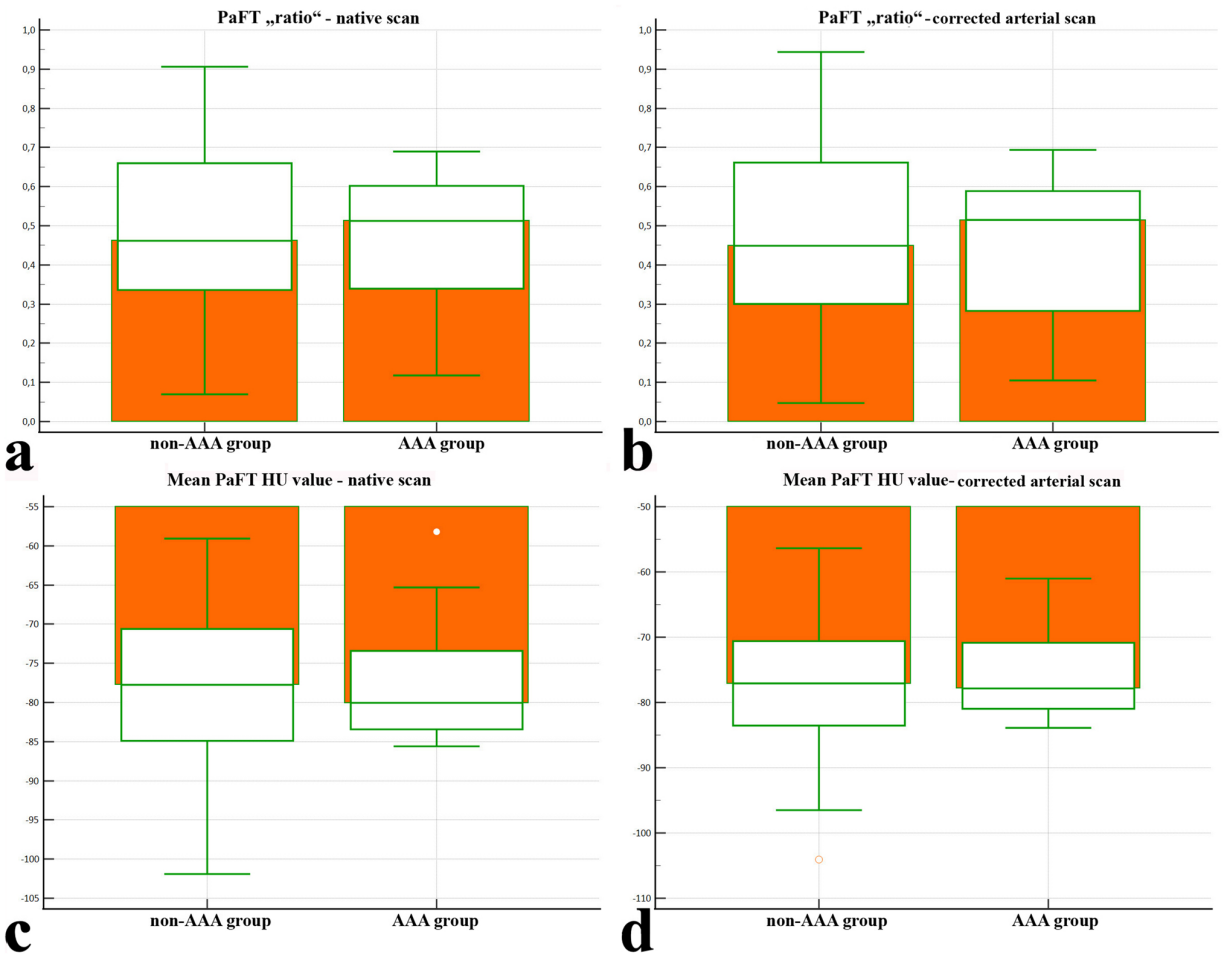
Secondary study results. Box–Whisker plots (medians) for PaFT “ratios” [PaFT volumes] in the non-AAA vs. AAA groups measured in native (0.462 vs. 0.513) (**a**) and corrected arterial (0.449 vs. 0.515) (**b**) scans, as well as for mean PaFT HU values in the native (− 77.7 vs. − 80.0) (**c**) and corrected arterial (− 77.0 vs. − 77.8) (**d**) scans. The Mann–Whitney test showed no significant difference between the non-AA and AAA groups

When comparing PaFT „ratio“ [PaFTVolume] and mean PaFT HU values in the pooled non- AAA and AAA groups in both native and arterial phases, no significant difference was found. The Mann–Whitney test produced corrected arterial PaFT median values that were almost identical to the native median values. Although a trend towards higher PaFT values in the AAA group was noticed, results were hampered by the low number of AAAs included. (Online Resource 9) (Fig. 4).

### Reproducibility study

The ICC [95% CI] for one observer was: 0.996 [0.991–0.999] for Aortic Volumes, 0.991 [0.978–0.996] for PaFT Volumes and 0.998 [0.995–0.999] for PaFT Mean HU. The values for two observers were: 0.986 [0.975–0.989], 0.980 [0.953–0.987] and 0.987 [0.974–0.989], respectively.

### Methodological considerations

**Fig. 5:**
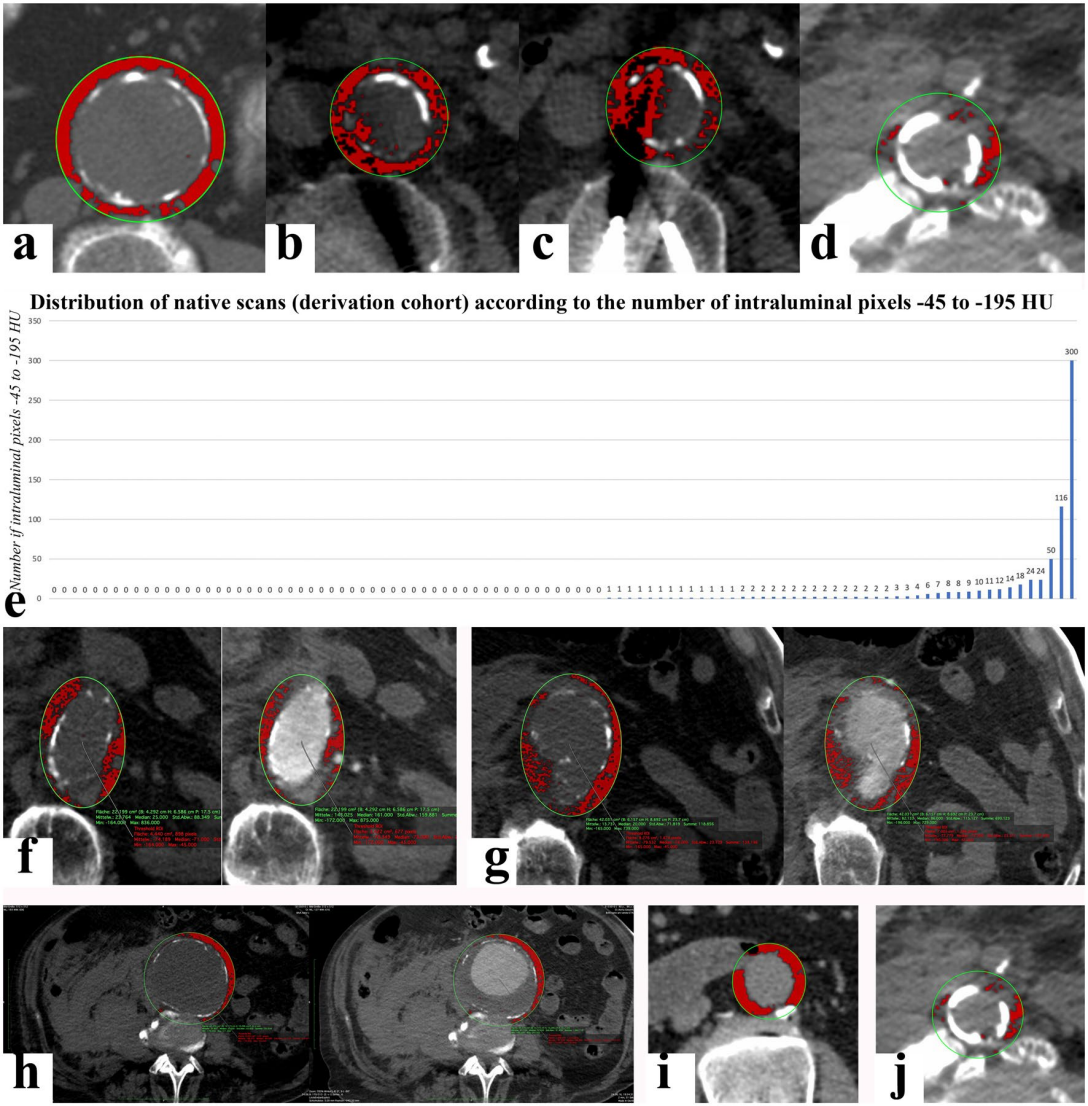
Methodological considerations/limitations. Presence of intraluminal pixels with values − 45 to − 195 HU in native CT scans. Usually, only solitary pixels in few axial CT images are seen (**a**). Such pixels appear in the vicinity of metal/high density foreign objects, for instance near spinal osteosynthesis materials (**b**, **c**). Alternatively, they can arise near very high-density calcium deposits in the aorta or vertebral column (**d**). Distribution of unenhanced CT scans of the derivation cohort with increasing number of intraluminal voxels with values of − 45 to − 195 HU (**e**). More than half of the CT-scans (55/101) had zero voxels, two-thirds (67/101) had 1 or less voxels, 83/101 had 2 or less voxels and 91/101 had 10 or less voxels. Of the 10 examinations with > 10 voxels the number of false positive voxels was still miniscule compared to the total number of fat-containing voxels (typically 1500–25,000). Considering the difference of fat-containing voxels between the unenhanced and enhanced data sets, the false positive voxels of these ten last examinations would account only for 0.27–2.67% of the measured difference in voxels − 45 HU to − 195 HU between the unenhanced and enhanced phases. Thus, the number of intraluminal voxels with fat tissue values in the aortic lumen of unenhanced scans does not warrant the exclusion of the unenhanced aortic disc when counting voxels with values − 45 to − 195 HU. Non-circular periaortic ROIs (**f**), irregular aortic disc shapes (**g**), periaortic hematoma (**h**) (blood and hematin-containing tissue in close proximity to the aortic wall will interfere with the detection of fat-containing voxels and para-aortic organs and foreign objects [intestinal segments can displace normal periaortic tissue and interfere with the measurement of PaFT (**i**) while artifacts from foreign objects can cause the loss of PaFT tissue voxels (**j**)] posed no limitation for our study, since identical segments of periaortic tissue were compared in the two CT phases. While para-aortic foreign tissue will interfere with PaFT Volume quantification, the mean PaFT HU value is not affected, since it is measured only in periaortic areas within the − 195 to − 45 HU range and not in the entire periaortic ring

Isolated pixels with values in the − 45 to − 195 HU range can be very rarely seen within the unenhanced aortic lumen, caused by high image noise or artifacts, e.g. metal/high-density foreign material artifacts. **(**Fig. 5a–d**)** To examine their impact and the possible need to exclude the aortic disc when measuring PaFT within the periaortic ROI, we counted their number in all native scans of the derivation cohort. Their number was so low that it did not warrant the exclusion of the aortic disc from the periaortic ROI. (Fig. 5e) Further methodological limitations like oval-shaped periaortic ROIs, irregular aortic contours, periaortic blood and organs in close vicinity to the aortic wall did not affect our results, since their effect was identical in both CT phases (Fig. 5f–j).

## Discussion

In our study, *PaFT Volumes* from the arterial and the native phase correlated almost perfectly (r > 0.99), as expected, while none of the eight potential confounding factors significantly affected the correlation. PaFT Volumes measured in the arterial phase were very constantly underestimated by about 10%. The reason for this could be either a very early enhancement of this tissue compartment, which is histologically characterized by an exceptionally rich vascularization [7, 24] or attenuation artifacts from intraluminal contrast medium [17, 18]. Because of the current CT-scanner resolution (600 microns), there are voxels adjacent to the wall containing both aortic wall and periaortic tissue, so that contrast enhancement of the aortic wall can also cause the average pixel HU value to exceed the − 45 HU limit. Corrected arterial PaFT Volumes showed very high agreement with native PaFT Volumes. *Mean PaFT HU values* in the arterial phase also showed a very high correlation (r > 0.95) with respective values measured in the native phase. The Mean HU value of PaFT seemed to remain constant (regression coefficient very close to 1.0) suggesting that the presence of intraluminal contrast medium does not significantly affect the Mean HU value of PaFT at all, as it has been shown for pericoronary fat [14]. This relation remained unaffected by the eight potential confounding factors examined. Corrected mean PaFT HU values, the agreed almost perfectly with respective values in native scans. Both *intra- and interobserver agreement* were very high. When comparing average PaFT values from the AAA and non-AAA groups in *the secondary study*, the corrected PaFT values from enhanced CT-scans showed very high agreement with those from unenhanced CT-scans.

Among studies focused on *PaFT Volume*, Schlett et al. measured abdominal PaFT Volume within 5 mm-wide coaxial periaortic rings. They examined, however, non-aneurysmatic aortas in unenhanced CT-scans [19]. Subsequently, other authors quantified PaFT in epidemiological studies (non-AAA related) but only in the thoracic aorta [7, 25–27]. Thanassoulis et al. found that higher PaFT Volumes correlate with larger aortic diameters [21]. However, they primarily correlated abdominal aortic dimensions with thoracic PaFT Volume as a proxy for abdominal PaFT, a confounding factor since thoracic PaFT is histologically different from abdominal PaFT [5]. When they correlated abdominal aortic dimensions with abdominal PaFT with the same result but they considered abdominal PaFT quantification less reliable [21]. However, they examined only non-enhanced CT scans and their sample was characterized by an underrepresentation of AAAs [21]. Dias-Neto et al. found no correlation of PaFT densities around AAAs with aortic diameter, although they found higher PaFT densities around the maximum AAA-diameter compared to the non-aneurysmatic infrarenal neck [22]. They compared, however, PaFT measurements of AAAs in enhanced CTA-scans to both enhanced and unenhanced CT-scans of the non-AAA control group [22]. To adjust PaFT Volume to aortic size, Dias-Neto determined the ratio of fat voxels area to the total area of the aortic disc in every axial image [22]. The Global Thresholding Plugin can measure the total volume of the ROIs with fat-containing voxels and then divide it with the total volume of the 5 mm-wide periaortic ring, greatly simplifying this step of the process.

Other studies focused on the *PaFT mean HU value* defined as the “fat attenuation index” [14, 23]. A recent study, for instance, measured the mean attenuation of abdominal periaortic fat volume [23]. This PaFT mean HU value seems to offer three distinct methodological advantages: it is unaffected by contrast medium and the presence of non-fatty periaortic tissue and does not require aortic size adjustment. It is unknown, however, which PaFT value (mean HU or Volume) is more representative of PaFT properties.

Regarding the separation of PaFT from retroperitoneal tissue, despite the close proximity of abdominal PaFT to other visceral fat tissues, PaFT is histologically clearly distinguished by small adipocytes and a rich capillary network [7]. Therefore, PaFT is morphologically and functionally a distinct entity, different from adjacent mesenterial and omental fat; however, the boundary between PaFT and visceral adipose tissue is not clear. For instance, Dias-Neto considered the potential confounding presence of mesenteric adipose tissue within the PaFT cylinder [22]. Existing histological evidence indicates that vascular wall inflammation (e.g. post-angioplasty) extends to at least several mm from the arterial wall [28] and pericardial fat around coronary arteries has a mean thickness of about 5 mm [17, 29]. Therefore, it seems reasonable to assume that PaFT extends to at least 5 mm from the aortic wall. The location of the retroperitoneal lining is also important, because adipose tissue on the other side of it can be included in the fat cylinder without having an effect on the aortic wall, since the two-fold membrane does not allow diffusion of secreted substances. These issues are important when defining a model for measuring PaFT, but not relevant for our primary study, whose objective is not to determine the histological limits of PaFT, but to determine whether contrast enhancement alters the CT characteristics of PaFT.

Study limitations were the low number of AAAs included, although our statistical analysis showed that our results were not affected by aortic size. The correction factors obtained in this retrospective, single center study should also be tested in a multi-center setting utilizing different scanners with different imaging parameters, as for instance we did not examine the effect of different contrast medium amounts and injection rates on PaFT values. The number of patients especially of the validation cohort was relatively low, yet equal to studies applying the same methodology [20]. Unlike most existing studies, we focused on the abdominal aorta, because of its clinical significance and the specific methodological challenges it poses for PaFT quantification. Excluded data sets were caused mostly by issues with the already reconstructed data sets (with no longer available raw data) and should be perceived as a limitation of the retrospective study and not the method itself. Other non-methodological issues beyond the scope of our study, like the position of the peritoneal lining, the width of the examined periaortic area or the effect of BMI and total abdominal fat tissue on the PaFT also need to be addressed further. Whereas our results were limited to the arterial phase, they are indicative of a constant effect of contrast medium on PaFT Volume and no effect on PaFT Mean HU value, which could also be the case for other non-arterial enhanced CT-phases as well.

## Conclusions

We demonstrated that PaFT Volume and mean HU value can be measured reliably and comparably in both unenhanced and enhanced scans, irrespective of intraluminal contrast densities, lateral or longitudinal contrast dispersion, extent of aortic wall calcification, aortic size or imaging parameters (slice thickness or CT-tube voltage). The aortic disc in the native phase does not need to be excluded from the measurement because of voxels with negative HU values. Volume measurements need to be standardized, e.g. per unit of aortic volume. The presence of other periaortic tissue can affect the total PaFT Volume but will not affect the mean PaFT HU value, since the latter is only determined by HU values within the area containing fat tissue and not the whole of the periaortic area. For PaFT quantification in axial images, the issue of non-circular ROIs has to be addressed and may necessitate different primary processing of raw data (unavailable in our study), excluding non-circular images or measuring PaFT only in certain locations (infrarenal neck or maximum diameter) of AAAs. The results of this methodological study will help establish the methodology to further elucidate the clinical importance and role of PaFT on vascular diseases (like AAAs) in additional studies.

### Supplementary Information

Below is the link to the electronic supplementary material.

Supplementary file1 (PDF 235 KB)

Supplementary file2 (PDF 329 KB)

Supplementary file3 (PDF 338 KB)

Supplementary file4 (PDF 521 KB)

Supplementary file5 (PDF 336 KB)

Supplementary file6 (PDF 464 KB)

Supplementary file7 (PDF 335 KB)

Supplementary file8 (PDF 317 KB)

Supplementary file9 (PDF 325 KB)

Supplementary file10 (PDF 408 KB)
