# Supplementary material for: Quantification of periaortic adipose tissue in contrast-enhanced CT angiography: technical feasibility and methodological considerations
Source: Int J Cardiovasc Imaging. 2022 Feb 26;38(7):1621–33. doi: 10.1007/s10554-022-02561-8 (PMC11142945; doi:10.1007/s10554-022-02561-8)
Supplement: Supplementary file 1 — Supplementary file1 (PDF 235 KB) [file 10554_2022_2561_MOESM1_ESM.pdf]

Quantification of periaortic adipose tissue in contrast-enhanced CT angiography: technical feasibility and methodological considerations

Original article

Short title: *quantification of periaortic fat in enhanced CT*

1. Apostolos T. Mamopoulos[a,b], MD **(**corresponding author**),** a.mamopoulos@web.de

Lutherplatz 40, 47805, Krefeld, Germany, Tel. 0049 170 5519575

2. Patrick Freyhardt[c,d] MD, PhD, patrick.freyhardt@helios-gesundheit.de

3. Aristotelis Touloumtzidis[b], MD aristotelis.touloumtzidis@helios-gesundheit.de

4. Alexander Zapenko[b], MD alexander.zapenko@helios-gesundheit.de

5. Marcus Katoh[a,c], MD, PhD marcus.katoh@helios-gesundheit.de

6. Gabor Gäbel[b], MD, PhD, gabor.gaebel@helios-gesundheit.de

[a] Faculty of Medicine, Saarland University, Kirrbergerstraße, D-66421 Homburg/Saar, Germany

[b] Department of Vascular Surgery, HELIOS Klinikum Krefeld
HELIOS Klinikum Krefeld, Lutherplatz 40, 47805, Krefeld, Germany

[c] Institute for diagnostic and interventional Radiology, HELIOS Klinikum Krefeld
HELIOS Klinikum Krefeld, Lutherplatz 40, 47805, Krefeld, Germany

[d] Faculty of Health, School of Medicine, University Witten/Herdecke, Witten
Universität Witten/Herdecke, Alfred-Herrhausen-Straße 50, 58455, Witten, Germany

Online Resource 1

Methodological Considerations

Non-circular aortic discs. There are a number of ways to deal with non-circular aortic discs in axial images. One approach is to reject them. ***Schlett** et al.* examined only nearly circular aortic discs by excluding subjects in whom the difference between transverse and anterior- posterior diameter remained >5 mm within the volume of interest because „the oval shape of the aorta precluded a standardized measurement of the periaortic adipose tissue cylinder." This is easier to achieve in shorter aortic segments (Schlett included only 40 mm long aortic segments) but becomes increasingly difficult when the entire infrarenal aortic segment is examined, where any angulation of the aorta would immediately exclude the patient. Secondly, nearly circular aortic discs are more ubiquitous in non-aneurysmatic aortas (examined by Schlett) and less so in AAAs. Another option we explored was to perform a stretched MPR reconstruction of the aortic segment and then generate axial images of the aorta orthogonally to the longitudinal axis of the reconstructed aorta. While the aortic disc is mostly nearly circular in the new axial images, the software can no longer perform the necessary volume reconstructions because of the variable slice thickness of the new, reconstructed axial images. Since the objective of this study was to explore the feasibility of PaFT measurement from contrast enhanced CT images compared to unenhanced CT images and not to establish a standard protocol for PaFT measurement, the standardization of the periaortic cylinder was not considered a priority. Therefore, we included non-circular aortic discs in our measurement by simply adjusting the aortic ROIs to match the contour of the aortic wall, ending up with a number of oval shaped aortic ROIs, identical in both CT phases.

Intraluminal voxels with negative HU values in the native scans. This approach assumes that all relevant voxels (-195 to -45 UH) in the periaortic ROI are located strictly within the 5 mm wide periaortic ring volume. One way to ensure this would be by excluding the volume within the aortic ROI, but since the aortic ROI cannot perfectly match the outer contour of the aortic wall, one risks excluding voxels that are in direct contact to the wall and thus very relevant for the measurement of PaFT. Experience with the method showed that no voxels within the -195 to -45 HU range were encountered in the aortic ROI of the arterial phase and very few/isolated if any such voxels were detected within the aortic disc in the unenhanced phase, so that by selecting the desired HU range within the periaortic ROI of both phases, one can safely assume that practically all -195 to -45 HU voxels are located within the 5 mm periaortic ring space.
