# Supplementary material for: Quantification of periaortic adipose tissue in contrast-enhanced CT angiography: technical feasibility and methodological considerations
Source: Int J Cardiovasc Imaging. 2022 Feb 26;38(7):1621–33. doi: 10.1007/s10554-022-02561-8 (PMC11142945; doi:10.1007/s10554-022-02561-8)
Supplement: Supplementary file 2 — Supplementary file2 (PDF 329 KB) [file 10554_2022_2561_MOESM2_ESM.pdf]

Quantification of periaortic adipose tissue in contrast-enhanced CT angiography: technical feasibility and methodological considerations

Original article

Short title: *quantification of periaortic fat in enhanced CT*

1. Apostolos T. Mamopoulos[a,b], MD **(corresponding author),** a.mamopoulos@web.de

Lutherplatz 40, 47805, Krefeld, Germany, Tel. 0049 170 5519575

2. Patrick Freyhardt[c,d] MD, PhD, patrick.freyhardt@helios-gesundheit.de

3. Aristotelis Touloumtzidis[b], MD aristotelis.touloumtzidis@helios-gesundheit.de

4. Alexander Zapenko[b], MD alexander.zapenko@helios-gesundheit.de

5. Marcus Katoh[a,c], MD, PhD marcus.katoh@helios-gesundheit.de

6. Gabor Gäbel[b], MD, PhD, gabor.gaebel@helios-gesundheit.de

[a] Faculty of Medicine, Saarland University, Kirrbergerstraße, D-66421 Homburg/Saar, Germany

[b] Department of Vascular Surgery, HELIOS Klinikum Krefeld
HELIOS Klinikum Krefeld, Lutherplatz 40, 47805, Krefeld, Germany

[c] Institute for diagnostic and interventional Radiology, HELIOS Klinikum Krefeld
HELIOS Klinikum Krefeld, Lutherplatz 40, 47805, Krefeld, Germany

[d] Faculty of Health, School of Medicine, University Witten/Herdecke, Witten
Universität Witten/Herdecke, Alfred-Herrhausen-Straße 50, 58455, Witten, Germany

Online Resource 2

author (year)	sample/vessel	method	range	ROI	software	CT-kV	Slice thickness
Schlett[a] (2009)	100 thoracic /100 abdominal aortas (no AAAs) -non enhanced CT	Manual selection / segmentation	-45 to -195 HU	Schlett protocol	Aquarius 3D, TeraRecon	120	2,5 mm
Fox[b] (2010)	1..205 patients throacic aorta -non enhanced CT	Manual selection and segmentation	-45 to -195 HU	Schlett protocol	Aquarius 3D, TeraRecon	120	2,5 mm
Lehman[c] (2010)	1067 thoracic aortas -non enahnced CT	Manual selection and segmentation	-45 to -195 HU	Schlett protocol	Aquarius 3D, TeraRecon	120	2,5 mm
Britton[d] (2012)	3.246 patients thoracic aorta -non enhanced CT	Manual selection/ segmentation	-45 to -195 HU	Schlett protocol	Aquarius 3D, TeraRecon	120	2.5 mm
Thanassoulis[e] (2012)	3001 patients for PAFT (thoracic aorta) for AAA size (abdom. /thoracic aorta) -non enhanced CT	Manual selection and segmentation	-45 to -195 HU	Schlett protocol	Aquarius 3D, TeraRecon	120	2,5 mm
Akyürek[f] (2014)	93 patients thoracic aorta	manual definition of ROIs	-200 to -450 HU	Schlett protocol	Volume Analysis Software, Siemens		
Efe[g] (2014)	323 patients thoracic aorta	Manual selection and segmentation	-50 to -200 HU	Schlett protocol	Argus, Siemens	120	
Maurovich-Horvat[h] (2015)	342 thoracic aortas	Manual selection and segmentation	-30 to -195 HU	Schlett protocol	Volume Viewer, Siemens	120	2,5 mm
Dias-Neto[i] (2018)	341 abdom. aortas 140: AAAs 104: stenotic aortas -enhanced CT 97: normal aortas -non enhanced CT	Manual selection and segmentation	-45 to -195 HU	Schlett protocol	OsirixMD	120	Thickness: variable 1-5mm Interval: 5-6mm

Online Resource 2. Published studies with PaFT quantification using computer tomography

a: Schlett CL, Bamberg F, Fox CS, Hoffmann U, Lehman SJ, Massaro JM, O'Donnell CJ (2009) Novel measurements of periaortic adipose tissue in comparison to anthropometric measures of obesity, and abdominal adipose tissue. Int J Obes (Lond) 33:226-232

b: Fox CS, Hoffmann U, Lehman SJ, Massaro JM, Meigs JB, Murabito JM, O'Donnell CJ, Schlett CL (2010) Peri-Aortic Fat Deposition Is Associated with Peripheral Arterial Disease: The Framingham Heart Study. Circ Cardiovasc Imaging 3:515-519

c: Lehman SJ, Fox CS, Hoffmann U, Massaro JM, O'Donnell CJ, Schlett CL (2010) Peri-aortic Fat, Cardiovascular Disease Risk Factors, and Aortic Calcification: The Framingham Heart Study. Atherosclerosis 210:656-661

d: Britton KA, Corsini EM, Fox CS, Hoffmann U, Massaro JM, Murabito JM, Pedley A (2012) Prevalence, Distribution, and Risk Factor Correlates of High Thoracic Periaortic Fat in the Framingham Heart Study. J Am Heart Assoc 1:e004200

e: Thanassoulis G, Corsini E, Fox CS, Hoffmann U, Massaro JM, Meigs JB, O'Donnell CJ, Rogers I, Schlett CL (2012) Periaortic Adipose Tissue and Aortic Dimensions in the Framingham Heart Study. J Am Heart Assoc 1:e000885

f: Akyürek Ö, Efe D, Kaya Z (2014) Thoracic periaortic adipose tissue in relation to cardiovascular risk in type 2 diabetes mellitus. Wien Klin Wochenschr 126:767–773

g: Efe D, Aygün F, Keser A, Ulucan Ş (2015) Relationship of Coronary Artery Disease with Pericardial and Periaortic Adipose Tissue and Their Volume Detected by MSCT. Hellenic J Cardiol 56:44-54

h: Maurovich-Horvat P, Engel LC, Hoffmann U, Kallianos K, Koenig W, Schlett CL, Szymonifka J, Truong QA (2015) Relationship of Thoracic Adipose Tissue Depots with Coronary Atherosclerosis and Circulating Inflammatory Biomarkers. Obesity (Silver Spring) 23:1178-1184

i: Dias-Neto M, Blankensteijn JD, Henriques-Coelho T, Hoozemans J, Lely RJ, Meekel JP, van Schaik TG, Sousa-Nunes F, Wisselink W, Yeung KK (2018) High Density of Periaortic Adipose Tissue in Abdominal Aortic Aneurysm. Eur J Vasc Endovasc Surg 56:663-671
