# Supplementary material for: Quantification of periaortic adipose tissue in contrast-enhanced CT angiography: technical feasibility and methodological considerations
Source: Int J Cardiovasc Imaging. 2022 Feb 26;38(7):1621–33. doi: 10.1007/s10554-022-02561-8 (PMC11142945; doi:10.1007/s10554-022-02561-8)
Supplement: Supplementary file 3 — Supplementary file3 (PDF 338 KB) [file 10554_2022_2561_MOESM3_ESM.pdf]

Quantification of periaortic adipose tissue in contrast-enhanced CT angiography: technical feasibility and methodological considerations

Original article

Short title: *quantification of periaortic fat in enhanced CT*

1. Apostolos T. Mamopoulos[a,b], MD **(**corresponding author**),** a.mamopoulos@web.de

Lutherplatz 40, 47805, Krefeld, Germany, Tel. 0049 170 5519575

2. Patrick Freyhardt[c,d] MD, PhD, patrick.freyhardt@helios-gesundheit.de

3. Aristotelis Touloumtzidis[b], MD aristotelis.touloumtzidis@helios-gesundheit.de

4. Alexander Zapenko[b], MD alexander.zapenko@helios-gesundheit.de

5. Marcus Katoh[a,c], MD, PhD marcus.katoh@helios-gesundheit.de

6. Gabor Gäbel[b], MD, PhD, gabor.gaebel@helios-gesundheit.de

[a] Faculty of Medicine, Saarland University, Kirrbergerstraße, D-66421 Homburg/Saar, Germany

[b] Department of Vascular Surgery, HELIOS Klinikum Krefeld
HELIOS Klinikum Krefeld, Lutherplatz 40, 47805, Krefeld, Germany

[c] Institute for diagnostic and interventional Radiology, HELIOS Klinikum Krefeld
HELIOS Klinikum Krefeld, Lutherplatz 40, 47805, Krefeld, Germany

[d] Faculty of Health, School of Medicine, University Witten/Herdecke, Witten
Universität Witten/Herdecke, Alfred-Herrhausen-Straße 50, 58455, Witten, Germany

Online Resource 3

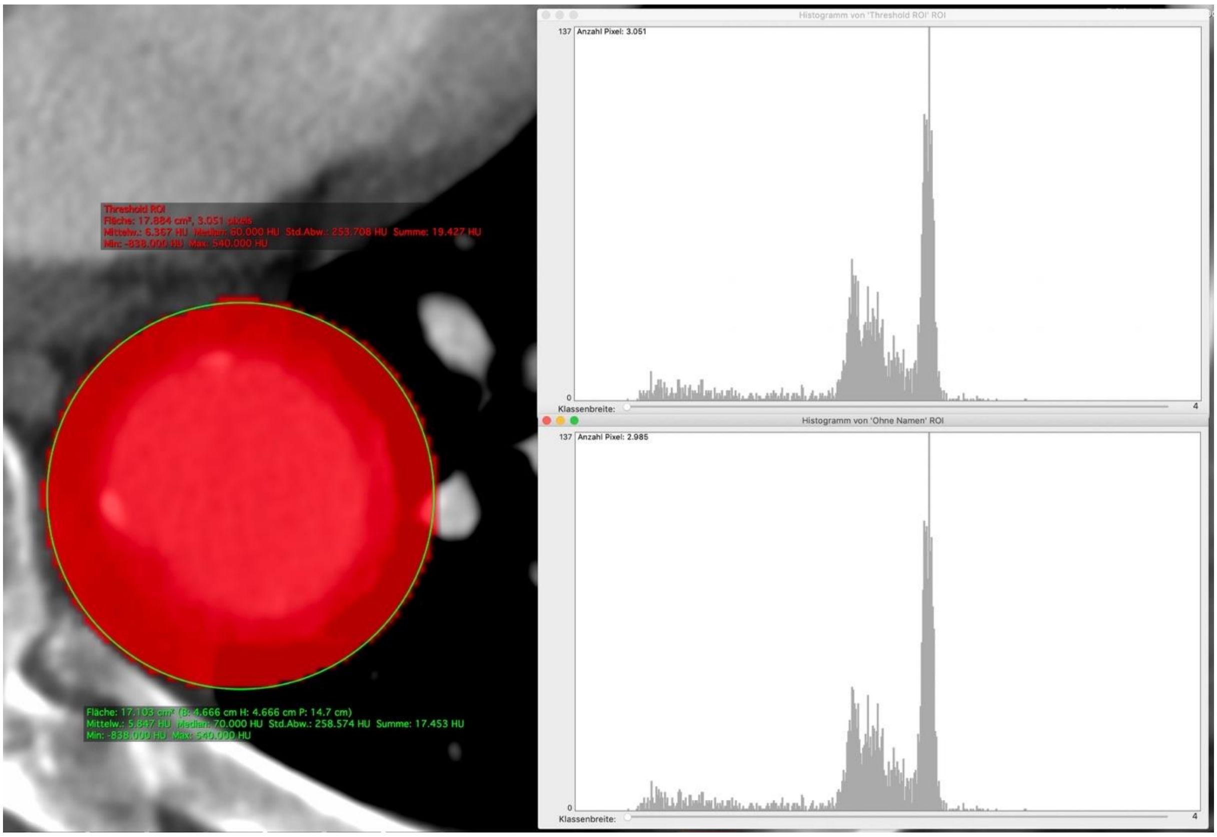

Online Resource 3. Independent Validation of the GolbalThresholding Plugin.

Before applying it, we independently validated the freely available, third-party GlobalThresholding Plugin. To do that, we set a periaortic ROI in both native and enhanced axial images using the OsiriXMD basic platform and then applied the GlobalThresholding Plugin set with the same pixel HU value range that was measured in the periaortic ROI. As demonstated in Figure X. both the basic values (ROI area in cm^2, Mean HU Values and their SD) and more importantly their histograms were identical for both the original ROI and the ROI created by summing all voxels with the Plugin. This provided independent validation of the correct function of the GobalThresholding Plugin and its ability to correctly identify all pixels within a given HU range and allocate their respective HU values.

This was achieved, by comparing the summary values (left image) and histograms (right image) of a standard periaortic ROI set with the basic OsiriX software and a ROI resulting from the addition of all pixels in the same HU range created with the Plugin. Both the total pixel areas, Mean HU values and their SD and the resulting histograms were identical for both ROIs, providing independent validation of the Plugin. Very small differences in the summary values were due to differences between OsiriX and the Plugin when allocating pixels on the very boundary of the ROI.
