# Supplementary material for: Quantification of periaortic adipose tissue in contrast-enhanced CT angiography: technical feasibility and methodological considerations
Source: Int J Cardiovasc Imaging. 2022 Feb 26;38(7):1621–33. doi: 10.1007/s10554-022-02561-8 (PMC11142945; doi:10.1007/s10554-022-02561-8)
Supplement: Supplementary file 8 — Supplementary file8 (PDF 317 KB) [file 10554_2022_2561_MOESM8_ESM.pdf]

Quantification of periaortic adipose tissue in contrast-enhanced CT angiography: technical feasibility and methodological considerations

Original article

Short title: *quantification of periaortic fat in enhanced CT*

1. Apostolos T. Mamopoulos[a,b], MD **(**corresponding author**),** a.mamopoulos@web.de

Lutherplatz 40, 47805, Krefeld, Germany, Tel. 0049 170 5519575

2. Patrick Freyhardt[c,d] MD, PhD, patrick.freyhardt@helios-gesundheit.de

3. Aristotelis Touloumtzidis[b], MD aristotelis.touloumtzidis@helios-gesundheit.de

4. Alexander Zapenko[b], MD alexander.zapenko@helios-gesundheit.de

5. Marcus Katoh[a,c], MD, PhD marcus.katoh@helios-gesundheit.de

6. Gabor Gäbel[b], MD, PhD, gabor.gaebel@helios-gesundheit.de

[a] Faculty of Medicine, Saarland University, Kirrbergerstraße, D-66421 Homburg/Saar, Germany

[b] Department of Vascular Surgery, HELIOS Klinikum Krefeld
HELIOS Klinikum Krefeld, Lutherplatz 40, 47805, Krefeld, Germany

[c] Institute for diagnostic and interventional Radiology, HELIOS Klinikum Krefeld
HELIOS Klinikum Krefeld, Lutherplatz 40, 47805, Krefeld, Germany

[d] Faculty of Health, School of Medicine, University Witten/Herdecke, Witten
Universität Witten/Herdecke, Alfred-Herrhausen-Straße 50, 58455, Witten, Germany

Online Resource 7

	PaFTVolume (with large AAAs)	PaFTVolume (without large AAAs)
**Sample size**	101	95
**equation**	**y = 1.1057 x**	**y = 1.1029 x**
**coefficient**	1.1057	1.1029
**Stand.error**	0.007136	0.007138
**95% CI**	1.0916 to 1.1199	1.0887 to 1.1171
**T**	154.9503	154.5169
**P**	< .0001	< .0001
**F-ratio**	24009.6103 P< .0001	23875.4738 P< .0001

	PaFTVolume (with large AAAs)	PaFTVolume (without large AAAs)
**Sample size**	101	95
**r^2**	.9921	.9881
**multiple r**	.9960	.9940
**Independent variables**		
**Arterial PaFT Volume**	P< .0001	P< .0001
**Agatston score**	P= .0716	P= .0912
**Mean contrast HU value**	P= .1952	P= .6280
**Aortic Diameter**	P= .3964	P= .6684
**Size of contrast sample-ROI**	P= .8338	P= .7395
**Slice thickness**	P= .1267	P= .7611
**Kilovoltage**	P= .7716	P= .8947
**Mean intraluminal SD, arterial phase**	P= .5286	P= .7894
**Longitidinal contrast variation**	P= .1462	P= .110

Online Resource 7. Univariate and multivariate regression analysis for PaFT Volumes from enhanced and unenhanced CT -scans.
