# Supplementary material for: Quantification of periaortic adipose tissue in contrast-enhanced CT angiography: technical feasibility and methodological considerations
Source: Int J Cardiovasc Imaging. 2022 Feb 26;38(7):1621–33. doi: 10.1007/s10554-022-02561-8 (PMC11142945; doi:10.1007/s10554-022-02561-8)
Supplement: Supplementary file 9 — Supplementary file9 (PDF 325 KB) [file 10554_2022_2561_MOESM9_ESM.pdf]

Quantification of periaortic adipose tissue in contrast-enhanced CT angiography: technical feasibility and methodological considerations

Original article

Short title: *quantification of periaortic fat in enhanced CT*

1. Apostolos T. Mamopoulos[a,b], MD **(corresponding author),** a.mamopoulos@web.de

Lutherplatz 40, 47805, Krefeld, Germany, Tel. 0049 170 5519575

2. Patrick Freyhardt[c,d] MD, PhD, patrick.freyhardt@helios-gesundheit.de

3. Aristotelis Touloumtzidis[b], MD aristotelis.touloumtzidis@helios-gesundheit.de

4. Alexander Zapenko[b], MD alexander.zapenko@helios-gesundheit.de

5. Marcus Katoh[a,c], MD, PhD marcus.katoh@helios-gesundheit.de

6. Gabor Gäbel[b], MD, PhD, gabor.gaebel@helios-gesundheit.de

[a] Faculty of Medicine, Saarland University, Kirrbergerstraße, D-66421 Homburg/Saar, Germany

[b] Department of Vascular Surgery, HELIOS Klinikum Krefeld
HELIOS Klinikum Krefeld, Lutherplatz 40, 47805, Krefeld, Germany

[c] Institute for diagnostic and interventional Radiology, HELIOS Klinikum Krefeld
HELIOS Klinikum Krefeld, Lutherplatz 40, 47805, Krefeld, Germany

[d] Faculty of Health, School of Medicine, University Witten/Herdecke, Witten
Universität Witten/Herdecke, Alfred-Herrhausen-Straße 50, 58455, Witten, Germany

Online Resource 8

	PaFTMeanHU (with large AAAs)	PaFTMeanHU (without large AAAs)
**Sample size**	101	95
**Equation**	**y = 1.0011 x**	**y = 0.9996 x**
**Coefficient**	1.0011	0.9996
**Stand.error**	0.004008	0.004081
**95% CI**	0.9932 to 1.0091	0.9915 to 1.0077
**T**	249.7950	244.9658
**P**	< .0001	< .0001
**F-ratio**	62397.53436 P< .0001	60008.23198 P< .0001

	PaFTMeanHU (with large AAAs)	PaFTMeanHU(without large AAAs)
**Sample size**	101	95
**r^2**	.9401	.9443
**multiple r**	.9696	.9718
**Independent variables**		
**Arterial PaFT mean HU value**	P< .0001	P< .0001
**Agatston score**	P= .2025	P= .059
**Mean contrast HU value**	P= .5968	P= .1379
**Aortic Diameter**	P= .5240	P= .2120
**Size of contrast sample-ROI**	P= .1996	P= .5023
**Slice thickness**	P= .2480	P= .0863
**Kilovoltage**	P= .1469	P= .0894
**Mean intraluminal SD, arterial phase**	P= .0805	P= .0856
**Longitudinal contrast variation**	P= .7602	P= .1098

Table 8. Univariate and multivariate regression analysis for PaFT Mean HU values from enhanced and unenhanced CT -scans.
