# Supplementary material for: Quantification of periaortic adipose tissue in contrast-enhanced CT angiography: technical feasibility and methodological considerations
Source: Int J Cardiovasc Imaging. 2022 Feb 26;38(7):1621–33. doi: 10.1007/s10554-022-02561-8 (PMC11142945; doi:10.1007/s10554-022-02561-8)
Supplement: Supplementary file 10 — Supplementary file10 (PDF 408 KB) [file 10554_2022_2561_MOESM10_ESM.pdf]

Quantification of periaortic adipose tissue in contrast-enhanced CT angiography: technical feasibility and methodological considerations

Original article

Short title: *quantification of periaortic fat in enhanced CT*

1. Apostolos T. Mamopoulos[a,b], MD **(**corresponding author**),** a.mamopoulos@web.de

Lutherplatz 40, 47805, Krefeld, Germany, Tel. 0049 170 5519575

2. Patrick Freyhardt[c,d] MD, PhD, patrick.freyhardt@helios-gesundheit.de

3. Aristotelis Touloumtzidis[b], MD aristotelis.touloumtzidis@helios-gesundheit.de

4. Alexander Zapenko[b], MD alexander.zapenko@helios-gesundheit.de

5. Marcus Katoh[a,c], MD, PhD marcus.katoh@helios-gesundheit.de

6. Gabor Gäbel[b], MD, PhD, gabor.gaebel@helios-gesundheit.de

[a] Faculty of Medicine, Saarland University, Kirrbergerstraße, D-66421 Homburg/Saar, Germany

[b] Department of Vascular Surgery, HELIOS Klinikum Krefeld
HELIOS Klinikum Krefeld, Lutherplatz 40, 47805, Krefeld, Germany

[c] Institute for diagnostic and interventional Radiology, HELIOS Klinikum Krefeld
HELIOS Klinikum Krefeld, Lutherplatz 40, 47805, Krefeld, Germany

[d] Faculty of Health, School of Medicine, University Witten/Herdecke, Witten
Universität Witten/Herdecke, Alfred-Herrhausen-Straße 50, 58455, Witten, Germany

Online Resource 9

Text 9. Results of the secondary study.

The *median of native [PaFTVolumes]*, in the non-AAA and AAA group was 0.462 (95% CI [0.405 – 0.543]) and 0.513 (95% CI [0.392 – 0.577] respectively, corresponding to a Hodges- Lehmann median difference of -0.00148 (95% CI [-0.116 to 0.102], which was not significant (n_{nonAAA}=122, n_{AAA}=19, U= 1 154, P= .976). The *median of corrected arterial [PaFT Volumes]* in the non-AAA and AAA group was 0.449 (95% CI [0.394 – 0.520]) and 0.515 (95% CI [0.343 – 0.579] respectively, corresponding to a Hodges-Lehmann median difference of -0.0101 (95% CI [-0.128 to 0.105], which was not significant (n_{nonAAA}=122, n_{AAA}=19, U= 1 124, P= .833).

The *median of native PaFT mean HU values* in the non-AAA and AAA group was -77.74 (95% CI [-79.91 to -74.08]) and -80.04 (95% CI [-83.01 to -73.68]) respectively, corresponding to a Hodges-Lehmann median difference of -0.334 (95% CI [-4.53 to 4.06]), which was not significant (n_{nonAAA}=122, n_{AAA}=19, U= 1 137, P= .894). The *median of corrected arterial PaFT HU mean values* in the non-AAA and AAA group was -77.07 (95% CI [-79.69 to -75.21]) and -77.84 (95% CI [-80.46 to -72.08]) respectively, corresponding to a Hodges-Lehmann median difference of 1.617 (95% CI [-2.7 to 5.56]), which was not significant (n_{nonAAA}=122, n_{AAA}=19, U= 1034, P= .450).

The indications for patients in the non-AAA group were: tumor diagnostic pancreas 17/122, kidney or urinary tract 22/122, hematoma detection in 25/122, as well as abdominal ache in 37/122 and diagnostic for abdominal/colorectal/gynecological complications 21/122.
